# Nitric Oxide and Hydrogen Sulfide Coordinately Reduce Glucose Sensitivity and Decrease Oxidative Stress via Ascorbate-Glutathione Cycle in Heat-Stressed Wheat (*Triticum aestivum* L.) Plants

**DOI:** 10.3390/antiox10010108

**Published:** 2021-01-14

**Authors:** Noushina Iqbal, Shahid Umar, Nafees A. Khan, Francisco J. Corpas

**Affiliations:** 1 Department of Botany, School of Chemical and Life Sciences, Jamia Hamdard, New Delhi 110062, India; sumer@jamiahamdard.ac.in; 2 Plant Physiology and Biochemistry Laboratory, Department of Botany, Aligarh Muslim University, Aligarh 202002, India; naf9.amu@gmail.com; 3 Group of Antioxidants, Free Radicals and Nitric Oxide in Biotechnology, Food and Agriculture, Department of Biochemistry, Cell and Molecular Biology of Plants, Estación Experimental del Zaidín, CSIC, Apartado 419, 18080 Granada, Spain

**Keywords:** AsA-GSH, glucose, H_2_S, nitric oxide, photosynthesis

## Abstract

The involvement of nitric oxide (NO) and hydrogen sulfide (H_2_S) in countermanding heat-inhibited photosynthetic features were studied in wheat (*Triticum aestivum* L.). Heat stress (HS) was employed at 40 °C after establishment for 6 h daily, and then plants were allowed to recover at 25 °C and grown for 30 days. Glucose (Glc) content increased under HS and repressed plant photosynthetic ability, but the application of sodium nitroprusside (SNP, as NO donor) either alone or with sodium hydrosulfide (NaHS, as H_2_S donor) reduced Glc-mediated photosynthetic suppression by enhancing ascorbate-glutathione (AsA-GSH) metabolism and antioxidant system, which reduced oxidative stress with decreased H_2_O_2_ and TBARS content. Oxidative stress reduction or inhibiting Glc repression was maximum with combined SNP and NaHS treatment, which was substantiated by 2-4-carboxyphenyl-4,4,5,5-tetramethylimidazoline-1-oxyl-3-oxide (cPTIO) and hypotaurine (HT), scavengers for NO and H_2_S, respectively. The scavenge of H_2_S reduced NO-mediated alleviation of HS suggesting of its downstream action in NO-mediated heat-tolerance. However, a simultaneous decrease of both (NO and H_2_S) led to higher Glc-mediated repression of photosynthesis and oxidative stress in terms of increased H_2_O_2_ content that was comparable to HS plants. Thus, NO and H_2_S cooperate to enhance photosynthesis under HS by reducing H_2_O_2_-induced oxidative stress and excess Glc-mediated photosynthetic suppression.

## 1. Introduction

Global temperature rise is an emerging issue for plant growth and development as it causes oxidative stress in plants affecting their physiology and metabolism. Heat stress leads to excess production of reactive oxygen species (ROS) that disturbs plant membrane lipids and alters protein structure and functions. The plant response to HS is highly conserved and involves a complex regulatory network of signalling molecules. Nitric oxide (NO) is an emerging signalling molecule that counterfeits heat-induced adverse effect. Although NO causes nitroso-oxidative stress, it is equally important as a signalling molecule that protects against abiotic stress. Supplementation of NO to wheat seeds under HS was reported to reduce oxidative stress by enhancing the methylglyoxal (MG) and antioxidative system [1]. Various studies unravel the role of NO in reducing stress [2,3].

Hydrogen sulfide (H_2_S) is another gaseous signalling molecule in plants which is involved in modulating defence responses together with affecting plant growth and development [4]. While NO regulates post-translational modification (PTM) through S-nitrosation, H_2_S signaling causes protein persulfidation, which modifies the structure, localization, and function of a target protein. Persulfidation refers to the addition of S to the thiol group of cysteine (-SH) in proteins modifying it into persulfide group (-SSH) [5]. Exposure of plants to abiotic stress increases the production of ROS causing oxidative stress, and this is followed by increased persulfidation or S-nitrosation, which acts as a protective mechanism against protein oxidative damage. H_2_S is reported to protect plants from heat stress [6,7] by regulating stomatal movement [8,9,10], photosynthesis [11], and antioxidative enzyme activities [12]. Because of the similarity of their physiological effects, H_2_S and NO crosstalk under different stresses are gaining attention [13,14,15,16,17,18]. Both are small uncharged molecules and can easily diffuse in inter- or intracellular spaces without the need of any carrier or transporter [16]. H_2_S interacts with hydrogen peroxide (H_2_O_2_), calcium (Ca^2+^), NO, and abscisic acid (ABA) to bring about a response under stress [5,18,19]. Both NO and H_2_S enhances the activity of the antioxidant enzymes by PTM [12,20]. Under heat stress in maize, both SNP and NaHS and GYY4137 (a H_2_S donor) decreased the electrolyte leakage and reduced the stress. NO was also found to increase H_2_S levels through increased formation of L-cysteine desulfhydrase (LCD, E.C. 4.4.1.1.). Besides, the combined application of SNP and H_2_S more efficiently reduced heat stress, which was eliminated by the application of H_2_S inhibitors and scavenger, suggesting that H_2_S may act as a downstream signalling agent in NO-mediated heat stress tolerance in maize seedlings [6]. However, H_2_S may act upstream or downstream of NO in the signalling cascade [18], and multifaceted connections occur between NO and H_2_S involved in various physiological functions and pathways [17]. The positive interaction between H_2_S and NO is reported in bermudagrass under cadmium (Cd) stress [21] in *Pisum sativum* under arsenate stress (AsV) [22], in maize seedling under chromium stress [23,24], and in wheat under Cd stress [25]. Both H_2_S and NO are reported to affect each other’s responses to modulate the plants’ antioxidant and other stress molecules. Both S-nitrosation and persulfidation are reported to regulate catalase (CAT) via interaction with thiol groups [26]. Shan et al. [27] reported that under water stress, H_2_S increases the production of NO, which enhances AsA-GSH cycle for water stress tolerance via decreasing the monodehydroascorbate (MDA) production and electrolyte leakage and increasing plant height and biomass. The previous exploration by [28] has shown that exogenous NO helped in enhancing the activity of AsA-GSH cycle through ascorbate peroxidase (APX), glutathione reductase (GR), dehydroascorbate reductase (DHAR), and monodehydroascorbate reductase (MDAR) [28]. Shan et al. [29] described the influence of NO in reducing glucose (Glc)-mediated photosynthetic repression under salt stress. Chen et al. [30] found that under water stress, Glc content increases, while supplementation of H_2_S decreased the Glc content. However, till now we do not find a study that correlates the role of NO and H_2_S in reducing Glc content under heat stress and alleviating photosynthesis by increasing the antioxidants and components of AsA-GSH cycle. Thus, the present study aims to evaluate the mechanistic approach adopted by wheat under heat stress to reduce photosynthetic suppression both due to oxidative stress and accumulated Glc.

## 2. Materials and Methods

### 2.1. Plant Material and Growth Conditions

Healthy seeds of wheat (*Triticum aestivum* L.) cultivar WH 542, winter wheat, were treated with 0.01% HgCl_2_ followed by washing with double distilled water to remove any adhered chemical after sterilization. Sterilized seeds were sown in truncated pots with a 14 cm diameter across the top and 8 cm across the bottom, with a 15 cm height filled with acid-washed sand that was purified according to Hewitt [31]. All the pots were placed in an environmental growth chamber (Khera KI-261) with day/night temperatures that was maintained at 25/18 °C, and 12 h photoperiod (PAR 300 µmol m^−2^ s^−1^), relative humidity of 65 ± 5%. Two plants were maintained in each pot, and it was saturated every alternate day with full-strength Hoagland’s nutrient solution (300 mL) in the morning. The temperature stress treatment was given by subjecting the plants for 6 h during the mid of the 12 h photoperiod to 40 °C daily (10 days after sowing, at 2–3 leaf emergence stage) for 15 day, and were then allowed to recover at optimum temperature (25 °C) and grown for the experimental period (5 days, total time-period was 30 days). The Hoagland nutrient solution was provided on alternate days in the morning. The control plants were maintained throughout the experimental growth period (30 days) at 25 °C. Sampling was done at 30 DAS.

A concentration of 100 μM SNP (as a NO donor) and 200 μM NaHS (as an H_2_S donor) was applied either alone or in combination with the foliage of plants in HS treated or non-treated plants with a hand sprayer at 15 days after sowing (DAS). A surfactant teepol (0.5%) was added with the control and NO and H_2_S treatments. Treatments were arranged in a complete randomized block design, and the number of replicates for each treatment were four (*n* = 4). A second set of experiments were performed to substantiate the result where inhibitors of NO and H_2_S were applied under heat stress. One hundred µM cPTIO was used as NO scavenger, and 200 µM HT as H_2_S inhibitor for verifying the results. The concentration of chemicals was determined based on the study of Kaya et al. [25]. They were applied on HS and non-stressed plants at 15 DAS together with surfactant teepol (0.5%).

### 2.2. Leaf Crude Extracts for Enzymatic Assays

In chilled mortar and pestle, freshly sampled leaves (200 mg) were homogenized with an extraction buffer containing 0.05% (*v*/*v*) Triton X-100 and 1% (*w*/*v*) PVP in potassium phosphate buffer (100 mM, pH 7.0). The homogenate was centrifuged for 20 min at 15,000× *g* and 4 °C. After centrifugation, the supernatant obtained was used for the activity assay of the different enzymes. The leaves that were taken for sampling were fully expanded and of the same age in each sampling. They were used directly for extraction.

### 2.3. Glucose (Glc) Content

Glucose content in leaf was determined adopting the method of Krishnaveni et al. [32], and Glc was used as a standard. Dried leaves were dissolved in 80% ethanol to obtain the leaf extract, which was heated in a water bath for 10 min at 60 °C and then allowed to cool. The samples were cooled and were then centrifuged at 1500× *g* for 1 min. The supernatant that was obtained was collected for the estimation of Glc content. The reaction mixture contained 1.0 mL of methanol, 25 mg O-dianisidine, 49 mL of phosphate buffer (0.1 M) at pH 6.5, peroxidase (5.0 mg), and 5.0 mg of Glc oxidase. To this reaction mixture (1.0 mL) 0.5 mL of the enzyme extract was added in a test tube to start the reaction. The test tubes were incubated at 35 °C, for 40 min. To terminate the reaction, 2.0 mL of 6 N HCl was added. At 540 nm, the colour intensity of the reaction mixture was recorded.

### 2.4. NO, H_2_S and H_2_O_2_ Content

For determination of NO content, nitrite content was estimated by adopting the method of Zhou et al. [33] with slight modifications. Fresh leaves (0.5 g) were taken and treated with 3.0 mL of ice-cold acetic acid buffer (50 mM, pH 3.6) having 4% zinc acetate. With the help of mortar and pestle, the leaf mixture was homogenized. The homogenate obtained was centrifuged for 15 min at 11,500× *g* and 4 °C. The supernatant was collected and pellets were washed with extraction buffer (1.0 mL), and then they were again centrifuged. Now, there were two supernatants from the two spins, and both were combined and were neutralized by the addition of 100 mg charcoal. Vortex and filtration were done, and the filtrate was leached and collected. In the ratio of 1:1, one ml for each of filtrate and Griess reagents (1.0% sulphanilamide and 0.1% -1-naphthyl ethylenediamine dihydrochloride in 0.5% H_2_PO_4_ solution) were mixed. The mixture was incubated at 540 nm and the content of NO was estimated from the calibration curve that was plotted using sodium nitrite as standard.

The content of leaf H_2_S was estimated by methylene blue formation from dimethyl-p-phenylenediamine in HCl as discussed by Xie et al. [34] with slight modification. In 0.7 g of leaves, 2.5 mL of Tris-HCl buffer (20 mM L^−1^, pH 6.8) containing 10 mM L^−1^ ethylene diamine tetraacetic acid (EDTA) was added, and the leaves were grounded in it. The homogenate obtained was centrifuged at 4 °C and 12,000× *g* for 15 min. In the supernatant (0.75 mL), 0.2 mL of 1% (*w/v*) zinc acetate was added for trapping H_2_S. It was allowed to develop for 30 min and then 0.1 mL of 20 mM L^−1^ dimethyl-p-phenylenediamine dissolved in 7.2 mol L^−1^ of HCl and 0.1 mL of 30 mM L^−1^ ferric chloride in 1.2 mol L^−1^ of HCl were added Spectrophotometrically at 670 nm, and the methylene blue formation was determined. Different concentrations of NaHS were used as standard curve expressed as nmol g^−1^ fresh weight (FW).

Leaf H_2_O_2_ content was determined by following Okuda [35]. Fresh leaves (500 mg) were ground in 200 mM of perchloric acid (HClO_4_) that was ice cold. The homogenate obtained was centrifuged at 1500× *g* for 10 min, and then HClO_4_ of the supernatant was neutralized with the addition of 4 M KOH. Insoluble KClO_4_ formed was eliminated by another centrifugation at 500× *g* for 3 min. The reaction mixture contained 1.5 mL of eluate, 80 μL of 3-methyl-2-benzothiazoline hydrazone, 400 μL of 12.5 mM 3-dimethyl aminobenzoic acid in 0.375 M phosphate buffer (pH 6.5), and 20 μL of peroxidase (0.25 unit). The reaction was started by the addition of peroxidase at 25 °C and at 590 nm, and the increase in absorbance was recorded.

### 2.5. Lipid Peroxidation

The content of thiobarbituric acid reactive substances (TBARS) was estimated by using the method of Dhindsa et al. [36], which provided the status of lipid peroxidation in leaves. To 500 mg of fresh leaves, 0.25% of 2-thiobarbituric acid (2-TBA) in 10% trichloroacetic acid (TCA) was added, and this mixture was heated for 30 min at 95 °C and then cooled rapidly on an ice bath. Centrifugation of the mixture was done at 10,000× *g* for 10 min. Supernatant (1 mL) was taken, and 4.0 mL of 20% TCA containing 5% TBA was added, and final colour intensity was recorded at 532 nm.

### 2.6. Non-Enzymatic Antioxidant Components

Reduced and oxidized glutathione (GSH and GSSG, respectively) was assayed through an enzymatic recycling method [37]. In this method, sequential oxidation by 5,5′-dithiobis-(2-nitrobenzoic acid) (DTNB) and reduction by NADPH was observed in the presence of glutathione reductase. The complete details for GSH and redox state determination was explained by Fatma et al. [38].

Ascorbic acid (AscA) and dehydroascorbate (DHA, corresponding to the oxidized ascorbate) were determined adopting the method of Law et al. [39] with slight modifications. To 500 mg of fresh leaves, 2 mL of potassium phosphate buffer (100 mM, pH 7.0) containing EDTA (1 mM) was added and then centrifuged at 10,000× *g* for 10 min. The supernatant obtained (1 mL) was mixed with 0.5 mL of 10% (*w*/*v*) TCA. It was thoroughly mixed, and was then incubated at 4 °C for 5 min. The 1.5 mL of the above solution and 0.5 mL of NaOH (0.1 M) were thoroughly mixed and were centrifuged at 5000× *g* at 20 °C for 10 min. The aliquot obtained after centrifugation was distributed equally in two separate 750 μL microfuge tubes. For AsA estimation, 200 μL of potassium phosphate buffer (150 mM, pH 7.4) was added to 750 μL of the aliquot, and for DHA estimation, 100 μL of DTT was added to 750 μL of the aliquot, followed by vortex-mixing and incubation for 15 min at 20 °C, followed by the addition of 100 μL of 0.5% (*w*/*v*) N-ethylmaleimide. Then, both microfuge tubes were incubated for 30 sec at room temperature. Then, 400 μL of 10% (*w*/*v*) TCA 400μL of 4% (*w*/*v*) of N′, N-dimethyl bipyridyl dye, 400 μL of H_3_PO_4_, and 200 μL of 3% (*w*/*v*) FeCl_3_ were added and thoroughly mixed in each sample tube. After 1 h incubation at 37 °C, the absorbance at 525 nm was recorded. For AsA, a standard curve in the range of 5–55 nmol was used for calibration. For DHA, the standard curve range was 1–5 nmol.

### 2.7. Enzymatic Antioxidant Systems

Superoxide dismutase (SOD, EC; 1.15.1.1) was estimated adopting Beyer and Fridovich [40] and Giannopolitis and Ries [41] method. The inhibition of photochemical reduction of nitro blue tetrazolium was monitored. Method of Aebi [42] was used for catalase (CAT, EC; 1.11.1.6) determination with slight modification. In this, H_2_O_2_ disappearance was monitored at 240 nm. The method of Foyer and Halliwell [43] was used for glutathione reductase (GR, EC; 1.6.4.2) estimation, and Glutathione-dependent oxidation of NADPH was monitored at 340 nm. Ascorbate peroxidase (APX, EC; 1.11.1.11) was measured by Nakano and Asada [44] method and the decrease in the absorbance of ascorbate at 290 nm was recorded. For this assay, the extraction buffer was supplemented with 2.0 mM ascorbate. Dehydroascorbate reductase (DHAR, EC; 1.8.5.1) activity was measured following the method of Foyer et al. [45]. The increase in absorbance at 265 nm due to the GSH dependent production of AsA was recorded. The reaction mixture contained potassium phosphate buffer (0.1 M, pH 6.2), 50–100 g of proteins and 2 mM GSH. DHA (1 mM) was added to start the reaction. One enzyme unit was equivalent to the production of nmol AsA/(g dry weight min). MDAR (EC, 1.6.5.4) activity was measured adopting the method of Hossain et al. [46]. In the reaction mixture 50 mM Tris-HCl buffer (pH 7.5), 2.5 mM AsA, 0.2 mM NADPH, 0.5 unit of ascorbate oxidase and enzyme solution, and the final volume was made 700 µL. Addition of ascorbate oxidase was done to begin the reaction and the activity was calculated from the change in absorbance at 340 nm, for 1 min, using an extinction coefficient of 6.2 mM^−1^ cm^−1^. Bradford’s [47] method was used for determining the protein concentration, and bovine serum albumin was used as standard.

### 2.8. Photosynthetic Parameters

The maximal PS II photochemical efficiency (variable fluorescence to maximal fluorescence; Fv/Fm) was determined with the help of chlorophyll fluorometer (Junior-PAM, Heinz Walz, Effeltrich, Germany). To obtain minimal fluorescence (Fo) and Fm, the plants were dark-adapted for 30 min. To obtain Fm, the Fo was measured during the weak measuring pulses (125 µmol m^−2^ s^−1^) and a saturating pulse (720 µmol m^−2^ s^−1^). The difference between Fo and Fm gave Fv. The quantum yield efficiency of PS II was represented by the ratio of Fv to Fm.

Stomatal conductance (gs), intercellular CO_2_ concentration (Ci) and net photosynthesis (PN) were measured at a light saturating intensity of 300 µmol m^−2^ s ^−1^ and 370 ± 5 µmol mol^−1^ atmospheric CO_2_ concentration in fully expanded uppermost leaves of plants in each treatment using Infra-Red Gas Analyzer (CI-340, Photosynthesis system, CID Bio-Science, USA). In all measurements, the conditions were same as supplied in the growth chamber.

The method of Usuda [48] was used for determination of Rubisco activity. NADH oxidation was monitored at 340 nm and at 30 °C, after the addition of enzyme extract to the reaction mixture., This oxidation resulted in the conversion of 3-phosphoglycerate to glycerol-3-phosphate. The enzyme was extracted by using chilled mortar and pestle for homogenization of leaf tissue (1.0 g) in ice-cold extraction buffer that comprised of 0.25 M Tris-HCl (pH 7.8), 0.05 mM MgCl_2_, 0.0025 mM EDTA, and 37.5 mg DTT. The homogenate obtained was centrifuged for 10 min at 10,000× *g* and 4 °C. After centrifugation, the supernatant obtained was used for enzyme assay. The reaction mixture contained 100 mM Tris-HCl (pH8.0), 40 mM NaHCO_3_, 0.2 mM NADH, 4.0 mM ATP, 5.0 mM DTT, 10 mM MgCl_2_, 0.2 mM EDTA, 1.0 U of glyceraldehydes-3-phosphodehydrogenase, and 1.0 U of 3-phosphoglycerate-kinase and 0.2 mM of ribulose1,5-bisphosphate.

### 2.9. Leaf Area and Dry Mass Determination

Plant dry mass was measured after uprooted the plants and then the root was washed gently under running tap water to remove any soil particles. The plant was then dried in an oven at 80 °C until it reached a constant weight. The dried plant samples were then weighed to know their dry mass on an electronic balance. Leaf area meter (LA211, Systronic, New Delhi, India) was used to measure the leaf area.

### 2.10. Statistical Analysis

Analysis of variance (ANOVA) was used for data analysis and SPSS 17.0 for windows was used. Data were presented as mean ± SE (*n* = 4). The least significant difference (LSD) was calculated for the significant data at *p* < 0.05. Bars that showed the same letter were not significantly different by LSD test at *p* < 0.05.

## 3. Results

We aim to understand the potential interrelationship between NO and H_2_S in the mechanism of protection against heat stress (HS). Wheat plants were exposed to 40 °C, and were also supplemented with different chemical compounds including 100 μM SNP (as NO donor) or 200 μM NaHS (as H_2_S donor) alone or combined. Accordingly, key parameters were analysed including growth and photosynthetic parameters, as well as elements of ROS and NO metabolism.

**Figure 1:**
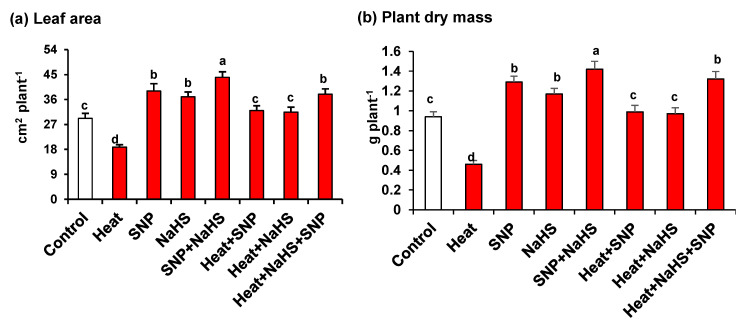
(**a**) Leaf area and (**b**) plant dry mass in wheat (*Triticum aestivum* L. var. WH 542) leaves treated with SNP (100 µM) and/or NaHS (200 µM) in the presence (40 °C) or absence (25 °C) of heat stress at 30 days after sowing (DAS). Data are presented as treatments mean ± SE (*n* = 4). Data followed by the same letter are not significantly different by least significant difference (LSD) test at (*p* < 0.05). SNP, sodium nitroprusside; NaHS, sodium hydrosulfide.

Figure 1 (panels a and b) shows that under HS, wheat plants are significantly affected, causing a substantial decrease of leaf area (36%) and plant dry mass (51%). On the other hand, the plants treated exogenously either with NO or with H_2_S, independently or in combination, both under optimal or stress conditions, the leaves presented significantly higher values of leaf area and dry mass. It is remarkable that the highest values in comparison to untreated plants were observed in plants grown under optimal conditions and treated only with SNP, but also in plants under HS treated simultaneously with NO and H_2_S where leaf area and dry mass increased by 30% and 40%, respectively.

**Figure 2:**
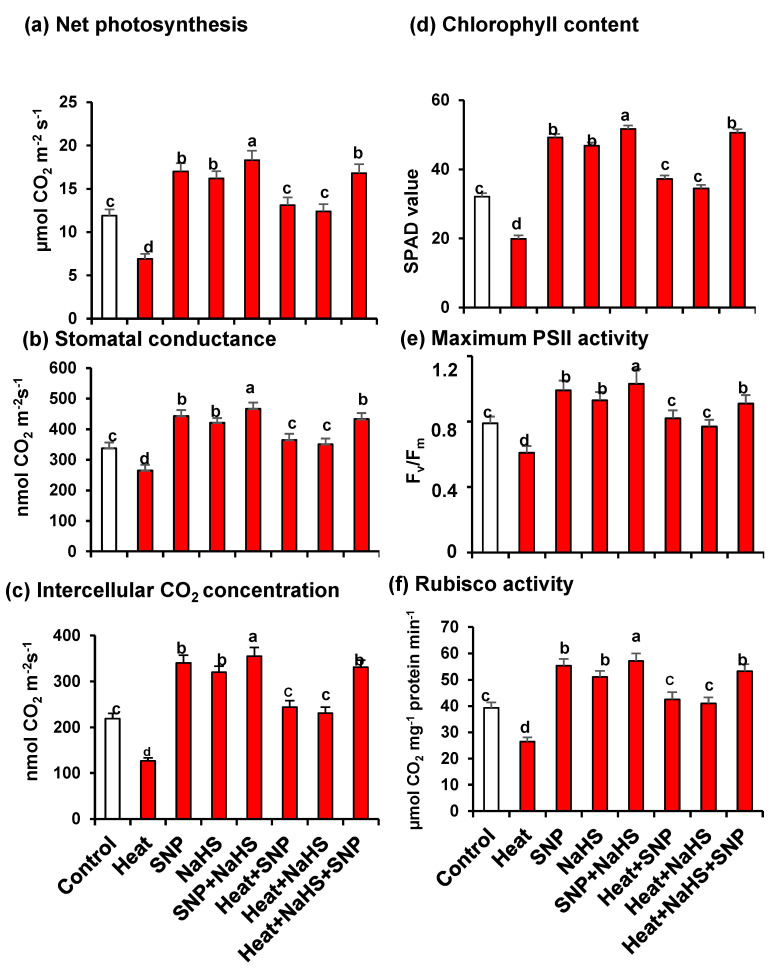
(**a**) Net photosynthesis. (**b**) Stomatal conductance. (**c**) Intercellular CO_2_ concentration. (**d**) Chlorophyll content. (**e**) Maximum PSII activity. (**f**) Rubisco activity in wheat (*Triticum aestivum* L. var. WH 542) leaves treated with SNP (100 µM) and/or NaHS (200 µM) in the presence (40 °C) or absence (25 °C) of heat stress at 30 days after sowing (DAS). Data are presented as treatments mean ± SE (*n* = 4). Data followed by the same letter are not significantly different by LSD test at (*p* < 0.05). SNP, sodium nitroprusside; NaHS, sodium hydrosulfide.

Figure 2 illustrates the analysis of Net photosynthesis (panel a), stomatal conductance (panel b), Intercellular CO_2_ concentration (panel c), chlorophyll content (panel d), maximum PSII activity (panel e), and Rubisco activity (panel f) in wheat leaves of untreated or heat-stressed plants treated or not with SNP (100 µM) and/or NaHS (200 µM) in alone or in combination. In all cases, these parameters have similar patterns because they were downregulated by HS, however, they experimented with a significant increase when the plants were treated exogenously either with NO or with H_2_S, independently or in combination, both under optimal conditions or under stress. Moreover, the highest values, in comparison to untreated plants, were observed in plants grown under optimal conditions treated only with SNP or NaHS, but also in plants under HS treated simultaneously with NO and H_2_S.

**Figure 3:**
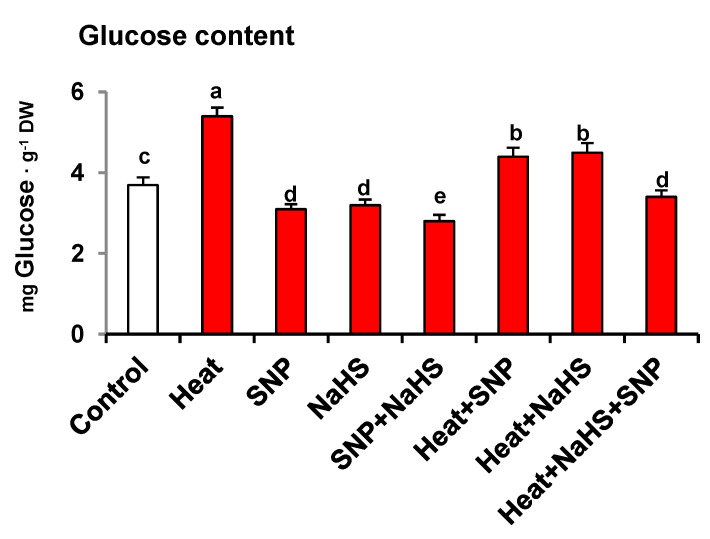
Glucose content in wheat (*Triticum aestivum* L. var. WH 542) leaves treated with SNP (100 µM) and/or NaHS (200 µM) in the presence (40 °C) or absence (25 °C) of heat stress at 30 days after sowing (DAS). Data are presented as treatments mean ± SE (*n* = 4). Data followed by the same letter are not significantly different by LSD test at (*p* < 0.05). DW, dry weight; SNP, sodium nitroprusside; NaHS, sodium hydrosulfide.

Figure 3 displays the glucose (Glc) content in wheat leaves of plants exposed to the same conditions described previously. Heat stress increased Glc content by 46%. However, the SNP and NaHS application caused a slight but significant decrease of Glc content by 16% and 14%, respectively, in comparison to the wheat plants under non-stress conditions. In the presence of HS, the decrease in Glc content by SNP and NaHS was significantly equal to each other compared to heat-stressed plant suggesting that both effectively lowered Glc content under HS. However, the combined application of SNP and NaHS decreased Glc content by 37% in comparison to heat-stressed plants.

**Figure 4:**
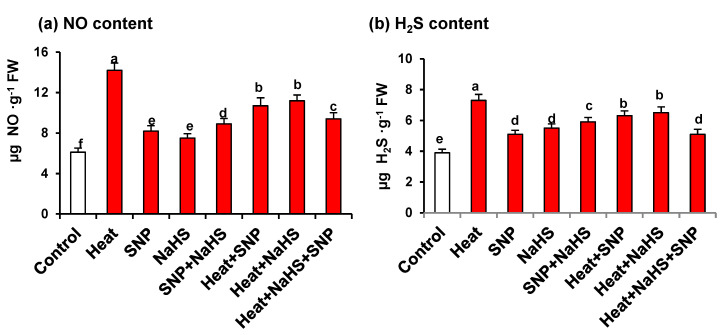
(**a**) Nitric oxide and (**b**) H_2_S content in wheat (*Triticum aestivum* L. var. WH 542) leaves treated with SNP (100 µM) and/or NaHS (200 µM) in the presence (40 °C) or absence (25 °C) of heat stress at 30 days after sowing (DAS). Data are presented as treatments mean ± SE (*n* = 4). Data followed by the same letter are not significantly different by LSD test at (*p* < 0.05). SNP, sodium nitroprusside; H2S, sodium hydrosulfide. FW, fresh weight.

Figure 4 shows the NO and H_2_S content in wheat leaves of plants exposed to the previously mentioned conditions. Both metabolites showed similar pattern with an increase in all the assayed conditions, in comparison with untreated plants. The highest values were observed under HS being the content of 2.3- and 1.9-fold higher for NO and H_2_S, respectively (Figure 4a,b). It is remarkable that under HS, plants treated with both NO and H_2_S showed lower values in the content of both molecules in comparison to plants treated only with one of these molecules.

**Figure 5:**
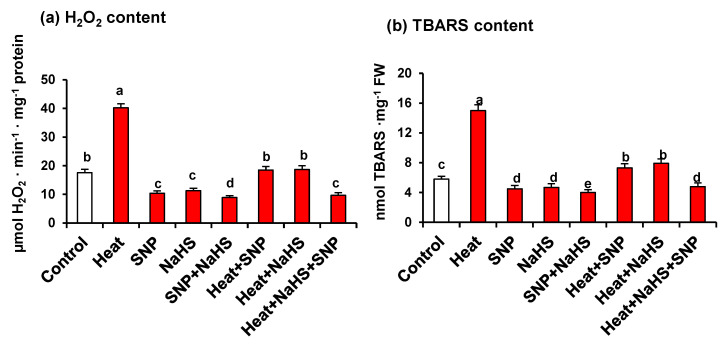
(**a**) H_2_O_2_ and (**b**) TBARS content in wheat (*Triticum aestivum* L. var. WH 542) leaves treated with SNP (100 µM) and/or NaHS (200 µM) in the presence (40 °C) or absence (25 °C) of heat stress at 30 days after sowing (DAS). Data are presented as treatments mean ± SE (*n* = 4). Data followed by the same letter are not significantly different by LSD test at (*p* < 0.05). SNP, sodium nitroprusside; NaHS, sodium hydrosulfide. FW, fresh weight.

Figure 5 displays the content of H_2_O_2_ (panel a) and TBARS (panel b), this last one being a recognized marker of oxidative damages of lipids. Both parameters show also a similar pattern against the different treatments described previously. Under HS, there is a significant increase, in comparison to untreated plants, being 2.3- and 2.5-fold higher for H_2_O_2_ and TBARS, respectively. The exogenous application of SNP or NaHS of heat stressed-plants triggers a drastic diminishing in the content of both H_2_O_2_ and TBARS. However, the simultaneous application of both compounds causes the maximum effects, which diminish the content of 76% and 68% for H_2_O_2_ and TBARS, respectively, in comparison to heat stressed-plants.

**Figure 6:**
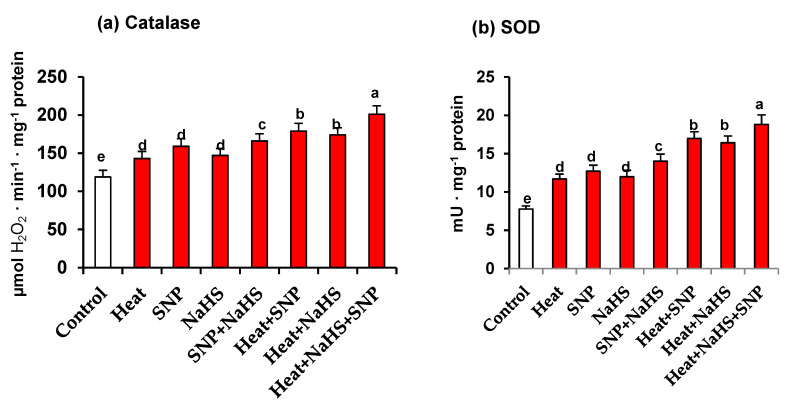
(**a**) Catalase activity and (**b**) superoxide dismutase (SOD) activity in wheat (Triticum aestivum L. var. WH 542) leaves treated with SNP (100 µM) and/or NaHS (200 µM) in the presence (40 °C) or absence (25 °C) of heat stress at 30 days after sowing (DAS). Data followed by the same letter are not significantly different by LSD test at (*p* < 0.05). SNP, sodium nitroprusside; NaHS, sodium hydrosulfide.

Catalase and SOD are considered the first line of defence against an increase of ROS. Figure 6 (panels a and b) show the activity of both enzymes in leaves of wheat plants exposed to the different conditions. Again, a similar pattern is observed in the activity of both antioxidant enzymes. In all the assayed experimental conditions, an increase in both enzymatic activities in comparison to unstressed wheat plants was found. However, the maximum activity increase was obtained in heat-stressed plants treated simultaneously, with NaHS and SNP being the activity higher at 69% and 142% for catalase and SOD, respectively, in comparison to control plants (Figure 6).

**Figure 7:**
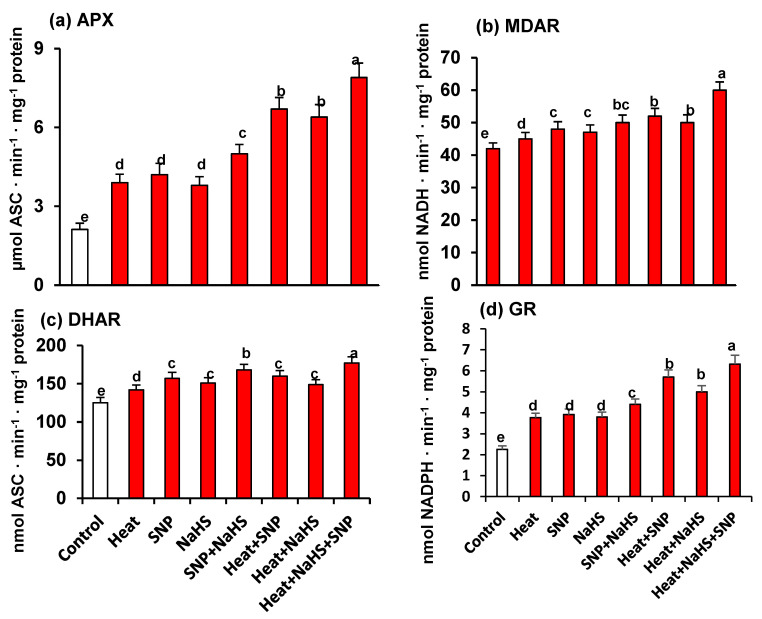
Activity of the ascorbate-glutathione cycle enzymes in wheat (*Triticum aestivum* L. var. WH 542) leaves treated with SNP (100 µM) and/or NaHS (200 µM) in the presence (40 °C) or absence (25 °C) of heat stress at 30 days after sowing (DAS). (**a**) Ascorbate peroxidase (APX) activity. (**b**) Monodehydroascorbate reductase (MDAR) activity. (**c**) Dehydroascorbate reductase (DHAR) activity. (**d**) Glutathione reductase (GR) activity. Data are presented as treatments mean ± SE (*n* = 4). Data followed by the same letter are not significantly different by LSD test at (*p* < 0.05). Asc, ascorbate; SNP, sodium nitroprusside; NaHS, sodium hydrosulfide.

**Figure 8:**
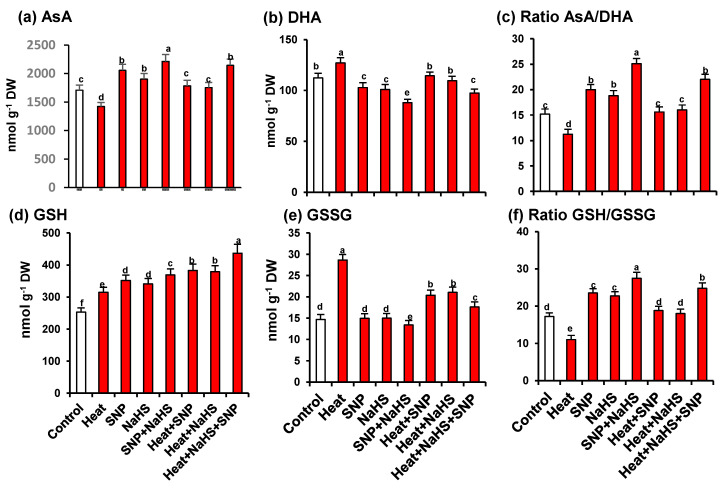
Content of non-enzymatic antioxidants of the ascorbate-glutathione cycle in leaves of wheat (*Triticum aestivum* L. var. WH 542) plants treated with SNP (100 µM) and/or NaHS (200 µM) in the presence (40 °C) or absence (25 °C) of heat stress at 30 days after sowing (DAS). (**a**) Ascorbate (AsA). (**b**) Dehydroascorbate (DHA). (**c**) Ratio AsA/DHA. (**d**) Reduced glutathione (GSH). (e) Oxidised glutathione (GSSG). (**f**) Ratio GSH/GSSG. Data are presented as treatments mean ± SE (n = 4). Data followed by same letter are not significantly different by LSD test at (*p* < 0.05). DW, dry weight. SNP, sodium nitroprusside. NaHS, sodium hydrosulfide.

As part of the analysis of the potential interrelationship between NO and H_2_S in the mechanism of tolerance of wheat plants under HS but the exogenous application of SNP and NaHS, all the enzymatic and non-enzymatic components of the ascorbate-glutathione cycle were also studied. Figure 7 shows the activity of APX (panel a), MDAR (panel b), DHAR (panel c), and GR (panel d). Similarly, to the observed profile of catalase and SOD activities, all the enzymatic components displayed an increase of its activity with all treatments in comparison to untreated wheat plants. However, the higher increase was observed in heat stressed-plants treated simultaneously with SNP and NaHS being this increase of 273% for APX, 39% for DHAR, 43% for MDAR, and 179% for GR. On the other hand, Figure 8 (panels a to f) illustrates the content of the non-enzymatic components of the ascorbate-glutathione cycle in wheat leaves. The AsA content was diminished by 17% under HS, but in the other experimental conditions showed a significant increase, being the leaves of stressed-plants treated simultaneously with SNP and NaHS where the AsA content was higher (26%) in comparison to unstressed plants. In the case of reduced GSH, the content was higher in all treatments in comparison to unstressed plants, being also the leaves of stressed-plants treated simultaneously with SNP and NaHS where the GSH content was higher (73%). In the case of the content of oxidized forms (DHA and GSSG), the pattern was similar. Thus, under HS, the DHA content showed a slight increase (13%), but in the case of GSSG content, the increase was very significant (195%). In the case of the ratio of AsA/DHA and GSH/GSSG, the pattern was similar with a significant decrease under HS, but with an increase in the other experimental conditions. Again, the maximum ratio of both was found in leaves of stressed-plants treated simultaneously with SNP and NaHS.

We found that the combined application of SNP and NaHS maximally alleviated HS, and this was because of the synergistic effect of NO and H_2_S on each other. However, application of cPTIO to SNP plus NaHS plus HS plants decreased the P_N_ by 7.4% compared to control. Supplementation of HT to the combined SNP and NaHS treatment under HS decreased P_N_ by 20.5%, which was greater than the decrease caused by cPTIO treatment, suggesting that the effect of NO was perhaps mediated via H_2_S. However, inhibitor of SNP and NaHS when applied together to SNP plus NaHS treated heat subjected plants resulted in a decreasing PN and brought it to the level of heat-treated plants, decreasing it by 35.2% compared to control.

The combined application of cPTIO and HT to SNP and NaHS treated HS plants reversed the effect of SNP and NaHS on plant growth and Glc content which was significantly equal to the HS plants, substantiating the coordinated role of NO and H_2_S in the protection of photosynthesis and growth via inhibiting Glc-mediated photosynthetic repression. Increased Glc content was decreased by both SNP and NaHS, but the application of their combined inhibitor reversed the alleviation and Glc-repression was again observed.

The decrease in H_2_O_2_ content that was observed in SNP and NaHS treated plants under HS was reversed when the inhibitor of SNP and NaHS was applied. Both cPTIO and HT when applied individually increased the H_2_O_2_ content compared to control. While cPTIO application brought the H_2_O_2_ level to control, it was HT application that increased it over control, but still it was below that of HS plants. However, on the combined application of HT and cPTIO, we observed that H_2_O_2_ increased significantly and came to the level of heat-stressed plants. Thus, both SNP and NaHS have their role in mitigating heat stress, but their action is rather dependent instead of being independent of each other (Table 1).

## 4. Discussion

Wheat is a staple food in the majority of the countries. It represents 30% of world grain production and contributes 50% to the world grain trade and 20% of calories consumed per capita [49]. It is adversely affected by heat and drought [50,51,52] and a decline of 4.1–6.4% in global wheat yield by the mid of 21st century is predicted due to increase in global temperature by 1 °C, while its demand, due to increasing population, is expected to increase by 60%. In India and China alone, a decrease of 8.0% and 3.0%, respectively, in wheat yield is expected due to a 1 °C rise in global mean temperature. Thus, temperature stress is hazardous for wheat growth, and measures should be adopted to reduce the impact of heat stress. Pan et al. [53] identified differential expression of several circular RNAs (circRNAs) in *Arabidopsis* under HS, and they differentially regulated the expression of defence-related genes through modulation of salicylic acid and NO signalling pathways. Expression of chloroplastic heat shock protein (Hsp) under HS was reported to protect the thermolabile photosystem II [54].

Among various signalling molecules, NO is gaining attention due to its effectiveness as a stress buster. It functions in heat stress signalling and acts upstream of AtCaM3 modulating HS transcription factor, DNA-binding activity, and Hsp accumulation [55]. However, the impact of NO as a potential signalling molecule depends on its concentration. NO and H_2_S are two important gaseous signalling molecules that regulate each other’s actions, and H_2_S might work upstream or downstream of NO depending upon its involvement in processes like stomatal closure or under abiotic stress, respectively [18]. In the present study, the impact of HS on wheat plants in terms of reduction in photosynthesis and growth and the involvement of NO and H_2_S in palliating the stress were analysed.

### 4.1. Heat Triggers Severe Stress and Glucose (Glc) Accumulation Which are Significantly Palliated by the Simultaneous Exogenous Application of NO and H_2_S

HS (40 °C) affects negatively wheat growth parameters (leaf area and dry mass) and reduces photosynthesis activity. However, all these parameters are significantly palliated by NO and H_2_S treatment, either when they were applied separately or together. However, when NO was supplemented with H_2_S maximum alleviation in photosynthetic parameters was found with increased content of Chl, Rubisco activity, maximum efficiency of PSII, P_N_, gs, and Ci. These results are in good agreement with previous ones, where NO increases photosynthetic rate and biomass of *Spinacia oleracea* [56] via affecting the chloroplast ultrastructure in flax leaf blades [57]. Ozfidan-Konakci et al. [58] found that in wheat plants under cobalt stress, exogenous NaHS or SNP regulates growth, Rubisco activity, water content, and AsA-GSH cycle.

Under heat stress accumulation of Glc was observed, which negatively influenced photosynthesis and growth. An early report showed that a high Glc accumulation leads to lower orthophosphate concentrations in the stroma, which may also lower the Rubisco activity [59]. Later, Bowes [60] showed that the carbohydrate accumulation triggered a decreased photosynthetic activity, either for the feedback inhibition of the RuBP/Pi regeneration capacities or by chloroplast disruption. Tholen et al. [61] reported that ethylene insensitivity resulted in greater Glc sensitivity, which negatively influenced Rubisco content and photosynthetic capacity of tobacco. In this study also, with an enhanced Glc level under HS, there was a reduction in photosynthesis with reduced Rubisco activity, but both exogenous NO and H_2_S helped in reducing this repression, probably by promoting growth via reduction of oxidative stress.

High sugar is reported to inhibit photosynthesis and growth, while at lower levels increase photosynthetic potential [62]. Thus, those signalling molecules that can divert the accumulated Glc for better utilization under stress so that feedback inhibition on photosynthesis is released is crucial for heat tolerance. In this regard, Sehar et al. [29] reported that NO reverses the photosynthetic repression by Glc under salt stress by reducing both Glc sensitivity and bringing the stress ethylene to an optimal level, which regulated stomatal conductance, photosynthetic activity, proline synthesis, and antioxidant metabolism to alleviate salt stress. Chen et al. [30] reported a decrease of Glc content in the NaHS-treated plants compared with the control plants both under drought and re-watering conditions.

Uchida et al. [63] reported that SNP supplementation increase the expression of sucrose-phosphate synthase and other stress-related genes that subsequently enhanced quantum yield for pigment system II and growth compared to control plants under salt stress. High temperature disturbs the chl biosynthesis and damages the photosynthetic apparatus by causing the grana stacks to swell up followed by ion leakage from leaf cells [64]. The increased Chl content in NO, H_2_S, and combined treatment are also responsible for increased photosynthesis in HS plants. Nitric oxide has been reported to enhance Chl biosynthesis and reduces Chl degradation [65]. This action of NO is dependent on its close relation to iron metabolism, which has a direct correlation with Chl biosynthesis and plant productivity. SNP is reported to promote the uptake, translocation, and internal availability of iron in plants [65,66,67]. Similar to NO, H_2_S also regulates photosynthesis under abiotic stress. Chen et al. [11] explored the role of H_2_S in photosynthesis regulation and found that H_2_S increases the Rubisco activity and modulates the expression of genes involved in photosynthesis and thiol redox modification. H_2_S modulates the expression of those genes which are involved in the process of photosynthate formation and thiol redox modifications, thus controlling photosynthesis [68].

Thus, we can summarise that similar to the reported studies on the photosynthetic repression by Glc, we found enhanced Glc accumulation under HS which was reduced by the application of either H_2_S and NO, but being higher this effect when they were applied simultaneously, suggesting the involvement of both molecules in decreasing Glc sensitivity and enhancing its utilization for promoting growth. Li et al. [69] reported that Glc is responsible for inducing stomatal closure, which is dependent on ROS and NO production. Several studies have shown growth and photosynthetic responses of plants under individual treatments of NO, H_2_S, and Glc under salt stress, but to our knowledge, the mechanism of interaction between how plants respond to the interactive effects of NO and H_2_S to reduce Glc accumulation for heat stress alleviation is new.

### 4.2. HS Triggers the Content of Endogenous NO and H_2_S

Previous reports indicate that under HS there is an increase in the endogenous content of either NO [69] or H_2_S [70]. In our experimental conditions, an increase of both NO and H_2_S content was also observed, which is in good agreement with these reports. Complementary studies have also suggested that the exogenous application of NO can increase heat tolerance [71]. Furthermore, the combined exogenous addition of NO and H_2_S synergistically boosts the synthesis and function of each other [13,18,72]. Kaya et al. [25] reported that under cadmium stress, both NO and H_2_S generation increases, and supplementation of H_2_S and NO exogenously further increases their content. Furthermore, the simultaneous application with the NO scavenger cPTIO with SNP or NaHS receiving plants significantly reduced NO generation and Cd tolerance, suggesting NO involvement in Cd-stress tolerance. However, application of the H_2_S scavenger hypotaurine to HS and NaHS receiving wheat plants reversed the NO generated with NaHS treatment, but was not as effective with SNP on endogenous NO levels.

Christou et al. [73] found that HS increases NO levels, and exogenous H_2_S donor results in inducing the accumulation of heat shock protein (HSP) in a NO-dependent manner. They reported that the exogenous application of either SNP or H_2_S increased NO generation in heat-stressed plants. The increase in NO generation with H_2_S suggests a positive relationship between them. Contrary to the above studies, in the present study, the maximum NO and H_2_S content was observed under HS, and supplementation of SNP and/or H_2_S decreased the production of NO and H_2_S. A similar decrease in NO content was reported under salt stress by NO supplementation compared to salt stress alone [29,74]. It is found that NO or H_2_S added to heat-treated plants decreased oxidative stress by increasing antioxidant metabolism. This resulted in lesser NO evolution in plants treated with heat and NO and/or H_2_S compared with HS alone. It has been reported that while at high concentrations, cellular production of NO causes extensive cellular damage, at low levels it is involved as a signal molecule in many important physiological processes [75]. On supplementation of SNP and NaHS, these molecules act as signalling molecules that activated the AsA-GSH cycle enzymes and antioxidative metabolism that scavenged the H_2_O_2_ content decreasing oxidative stress. When the oxidative stress was decreased, the content of NO and H_2_S also decreased. In fact, the homeostasis of NO/RNS has been reported to play an important role in plants acclimation to HS by neutralizing the excessive oxidative and nitrosative injury in plants. Thus, NO overproduction or S-nitrosothiols (SNOs) accumulation seems to be associated with increased heat sensitivity, due to nitro-oxidative stress, lipid peroxidation, protein tyrosine nitration, and oxidative damage to proteins and nucleic acids [76,77,78,79]. Therefore, NO dose is an important factor regarding its ameliorative property. The collected data show that endogenous NO accumulates under biotic and abiotic stresses, where NO is considered as an important signalling molecule and as a major tolerance factor against oxidative stress [33,80]. The increase in NO by SNP supplementation is expected, but NaHS supplementation also increased NO formation. Similarly, SNP increased H_2_S formation under HS. It was the combined application of H_2_S and NO that caused maximum NO and H_2_S generation. Ref. [25] reported that perhaps H_2_S increases NO synthesis and in turn is tightly regulated by NO. All these data support the close relationship between NO and H_2_S.

### 4.3. NO and H_2_S Trigger a General Increase of Antioxidant System Which Palliate the Oxidative Stress induced by HS

AsA-GSH cycle plays an important role in the scavenging of H_2_O_2_ and reducing oxidative stress [27]. In wheat plants subjected to HS, the activity of all enzymatic components of AsA-GSH cycle increased. However, the stimulation of this antioxidant system was not sufficient to palliate the HS induced-oxidative stress. Combined supplementation of NO and H_2_S under heat stress maximally enhanced the AsA-GSH metabolism together with the activity of CAT and SOD. This resulted in the scavenging of H_2_O_2_ to reduce oxidative stress. Tiwari and Yadav [81] reported the importance of the AsA–GSH system in managing redox metabolism under HS in maize. They suggested that the enzymatic and non-enzymatic component of AsA-GSH cycle play a key role in ROS homeostasis in cells, thus minimizing the oxidative stress in plants. Among the maize genotypes, the tolerant genotype showed better adaptability to HS due to enhanced activity of the enzymes of AsA-GSH cycle. Khan et al. [82] reported that NO-induced H_2_S alleviated the negative effects of osmotic stress on wheat seedlings by enhancing the activities of APX and GR. Both endogenous NO and exogenous NO were equally effective in regulating the activity of AsA-GSH cycle in *Agropyron cristatum* [83]. In wheat seedlings, H_2_S acted upstream of NO in the regulation of APX and GR activity by water stress [27]. Shan et al. [84] reported that in wheat seedlings under heat stress, exogenous H_2_S alleviated oxidative damage by regulating the AsA-GSH cycle.

In the AsA-GSH cycle, APX functions to oxidise AsA into MDA, which is disproportionate into AsA and DHA. The DHA formed is reduced to AsA by the enzyme DHAR which accepts an electron from two molecules of GSH, converting it into GSSG. Thus, the reduction of DHA to AsA occurs via oxidation of GSH to GSSG. There is the occurring interplay between the reduced and oxidised form that is essential to maintain the redox state in the cell. The GSSG, the oxidised form of GSH, has to be reduced to maintain the redox balance, and GR plays the mediator enzyme in this function of reducing GSSG to GSH by an NADPH-dependent reaction. Higher AsA and GSH content with reduced DHA and GSSG content with greater DHAR and GR activities help to maintain the reduced GSH level and redox state (GSH/GSSG, AsA/DHA) with combined NO and H_2_S treatment [85]. The enzyme APX has been reported to undergo PTM by both *S*-nitrosation and persulfidation, which increases the activity of this enzyme [12,18,86]. Here, the enhanced activity of APX was observed by NO and H_2_S, and maximally by their combined application. Besides APX, catalase is also subjected to PTM, both *S*-nitrosation and persulfidation [26,87]. Both NO and H_2_S inhibited CAT activity and complemented or antagonized each other in regulating H_2_O_2_ content by regulating the antioxidant enzymes [25]. Increased SOD activity with combined NO and H_2_S treatment reduced the H_2_O_2_ content together with APX and CAT activity. Contradictory to the present study, the combination of NaHS and SNP were not able to reduce lipid peroxidation more than their individual treatment in Cd-stressed bermudagrass [21].

H_2_S and NO both are involved in reducing oxidative stress and in combination, they maximally alleviated stress. Both H_2_O_2_ and TBARS content that were increased under HS were significantly and equally reduced by supplementation of either NO or NaHS to HS treated plants, however, the combined application of NO with H_2_S under HS maximally reduced their content. This shows the potentiality of combined treatment in stress mitigation. Antioxidant enzymes such as SOD, CAT, guaiacol peroxidase, and enzymes of the AsA-GSH cycle play a major role in detoxifying free radicals and H_2_O_2_ production during stress [88]. Valivand and Amooaghaie [89] reported that NaHS supplementation to *Cucurbita pepo* L. exposed to Ni stress reduced the electrolyte leakage and H_2_O_2_ content. Min et al. [90] reported that under HS, in wheat seedlings, application of NaHS reduced the heat-induced oxidative stress by lowering H_2_O_2_ and MDA content and enhancing the antioxidative enzymes. In rice exposed to HS, SNP application was capable of reducing the oxidative stress by decreasing H_2_O_2_ and electrolyte leakage content and enhancing photosynthetic potential [91]. It has been reported that when NO is supplemented with H_2_S, it alleviated the heat-stress induced oxidative stress by reducing ROS generation, which was more efficient than the NO single treatment [6]. It is remarkable that under HS all the wheat enzymatic antioxidant systems (SOD, catalase, and ascorbate glutathione cycle) were upregulated to palliate the oxidative damage. However, the exogenous application of both NO and H_2_S reinforced the upregulation of these antioxidant systems supporting the beneficial effects of these compounds. It is well known that NO and H_2_S can exert a regulatory function of proteins through PTMs such as tyrosine nitration, S-nitrosation, and persulfidation. Multiple reports are showing that some of these PTMs can exert either up- or downregulation in the activity of these proteins. For example, APX activity is upregulated by S-nitrosation and persulfidation [12,86], but it is downregulated by tyrosine nitration. However, catalase activity is downregulated by these three PTMs mediated by NO and H_2_S [26,92]. This information could be contradictory in part with the results found in our experimental conditions, however, it should be remarked that these studies were done under in vitro conditions using the purified proteins without a cellular environment. In the cells, redox conditions are more complete and the thiol group of cysteine could be targeted by different PTMs including S-nitrosation, persulfidation, S-glutathionylation, or sulfenation that can compete with each other depending on the cellular redox state of the cell [20]. Therefore, it could be difficult to compare results obtained under in vitro condition with that obtained in whole plants where multiples factors are involved.

## 5. Conclusions

**Figure 9:**
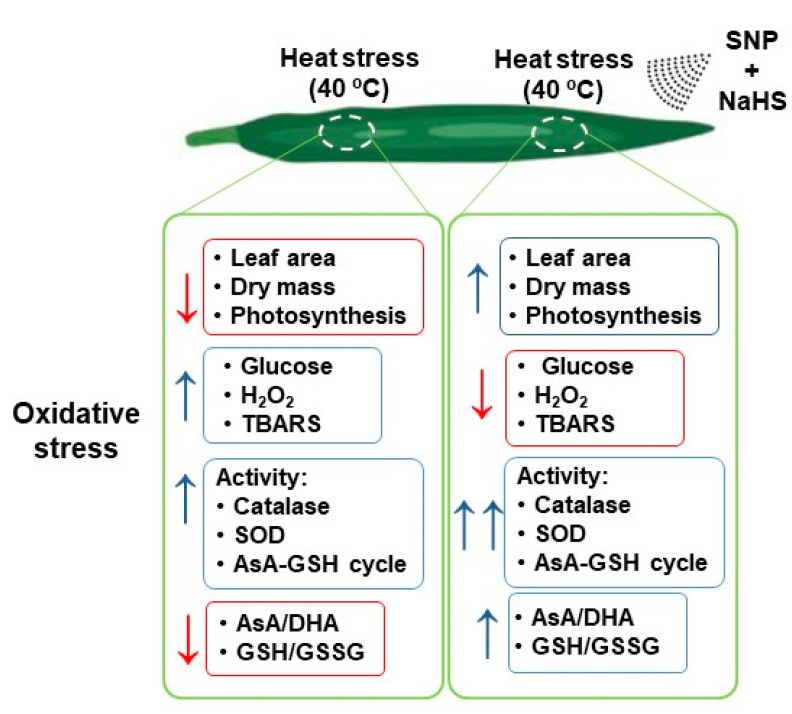
Summary of the main effects of HS (40 °C) in wheat leaf plants untreated or treated exogenously with SNP (100 µM) and NaHS (200 µM) as NO and H_2_S donors, respectively, which palliates the oxidative stress damages.

Heat stress is detrimental to plant photosynthesis and growth due to a ROS over-generation that disturbs plants’ redox homeostasis. Figure 9 displays a summary of the main negative effects triggered by HS in wheat leaves and how the exogenous application of NO and H_2_S can reverse/palliate these damages. The simultaneous exogenous application of NO and H_2_S significantly palliate the negative effects of HS by enhancing AsA-GSH metabolism. The AsA-GSH cycle effectively detoxifies excess H_2_O_2_ produced during HS and enhances the AsA/DHA and GSH/GSSG ratio to maintain the cellular redox state. It also functions in reducing the Glc-mediated photosynthetic repression. Under HS, excess Glc reduces photosynthesis by various mechanisms including negative feedback mechanism and stomatal closure. Under HS, both NO and Glc can induce stomatal closure and supplementation of NO as SNP reduced the stress NO content to reduce Glc-mediated stomatal closure and photosynthesis. Therefore, it could be concluded that the action of the exogenous NO is mediated via H_2_S under HS for promoting photosynthesis and growth, and their combined application is beneficial in combating heat stress.

## Figures and Tables

**Table 1:** H_2_O_2_ content (nmol g^−1^ FW), Glc (glucose content, mg g^−1^ DW), P_N_ (net photosynthesis, µmol CO_2_ m^−2^ s^−1^), and PDM (plant dry mass, g plant^−1^) in leaves of wheat (*Triticum aestivum* L. var. WH 542) plants treated with SNP (100 µM) and/or NaHS (200 µM) in the presence (40 °C) or absence (25 °C) of heat stress at 30 days after sowing (DAS). Data are presented as treatments mean ± SE (*n* = 4). Data followed by same letter are not significantly different by LSD test at (*p* < 0.05). cPTIO, 2-4-carboxyphenyl-4,4,5,5-tetramethylimidazoline-1-oxyl-3-oxide. DW, dry weight. H_2_O_2_, hydrogen peroxide. HT, hypotaurine. SNP, sodium nitroprusside. NaHS, sodium hydrosulfide.

Treatment	H_2_O_2_	Glc	P_N_	PDM
Control	17.5 ± 0.51 c	3.7 ± 0.18 c	12.2 ± 0.791 b	0.97 ± 0.031 b
Heat stress	39.4 ± 0.96 a	5.9 ± 0.25 a	7.1± 0.574 d	0.44 ± 0.017 d
Heat stress + SNP + NaHS	9.3 ± 0.36 d	3.0 ± 0.14 d	18.2 ± 1.102 a	1.61 ± 0.039 a
Heat stress + SNP + NaHS + cPTIO	19.8 ± 0.68 c	3.9 ± 0.19 c	11.3 ± 0.684 b	0.82 ± 0.025 b
Heat stress + SNP + NaHS + HT	30.5 ± 0.81 b	4.8 ± 0. 21 b	9.7± 0.629 c	0.64 ± 0.191 c
Heat stress + SNP + NaHS + cPTIO + HT	41.6 ± 0.99 a	5.7 ± 0.24 a	7.9± 0.628 d	0.49 ± 0.018 d

## Data Availability

The data presented in this study are available in the graph and tables provided in the manuscript.
